# Climate change knowledge is associated with greater worry but less anxiety about climate change

**DOI:** 10.1016/j.joclim.2026.100712

**Published:** 2026-07-22

**Authors:** Clara Kühner, Hannes Zacher

**Affiliations:** Wilhelm Wundt Institute of Psychology, Leipzig University, Neumarkt 9-19, 04109 Leipzig, Germany

**Keywords:** Climate change knowledge, Climate change anxiety, Climate change worry, Multilevel analysis

## Abstract

**Background:**

As climate change increasingly threatens human health and well-being, a growing number of individuals experience climate change anxiety. Whereas the role of certain personal characteristics, such as environmental attitudes, in climate change anxiety is well established, the association between climate change knowledge and climate change anxiety is currently not well understood, and previous studies produced mixed findings. The present study examines the unique associations between climate change knowledge and both climate change anxiety and climate change worry at the between- and within-person levels of analysis.

**Methods:**

We analyzed four waves of data collected across nine months from 1,152 adults in Germany using mixed-effects models. Climate change knowledge was assessed using two validated tests.

**Results:**

At the between-person level, climate change knowledge was positively associated with climate change worry but negatively associated with climate change anxiety. These associations were consistent across both knowledge tests and both subdimensions of climate change anxiety. At the within-person level, no significant associations emerged, except for a positive association between climate change knowledge and climate change worry for one of the two knowledge tests.

**Conclusion:**

These findings help reconcile previous inconsistent results by indicating that greater climate change knowledge may foster adaptive worry while simultaneously mitigating more clinical and impairing forms of climate change anxiety.

## 1 Introduction

Climate change and its effects, such as extreme weather, rising sea levels, and shortages of food and water, threaten human health and long-term survival [1]. In response to this threat, people increasingly experience *climate change anxiety*, defined as anxiety related to climate change [2,3]. For example, in a study with over 10,000 participants from 32 countries, 28.2% indicated that they were “very or extremely anxious” about climate change [4]. Climate change anxiety is associated with increased pro-environmental behavior, but also with impaired well-being [[5], [6], [7], [8], [9]]. It is therefore important to understand the factors that may predict climate change anxiety.

So far, research has primarily examined how certain personal characteristics such as environmental attitudes and climate change-related experiences are associated with climate change anxiety [5,10,11]. However, it is currently not well understood how *climate change knowledge*, defined as understanding of the physical underpinnings as well as causes and consequences of climate change [12,13], is related to climate change anxiety. This is an important oversight, because research suggests that climate change knowledge may increase risk perceptions and, in turn, climate change anxiety [14]. At the same time, many interventions aimed at promoting pro-environmental behavior rely on some form of knowledge dissemination [15], and climate change knowledge is also conveyed in educational settings (e.g., in schools and universities) and through the media (e.g., news coverage and articles). Accordingly, a better understanding of how climate change knowledge relates to climate change anxiety is needed, as these emotional responses may both motivate pro-environmental behavior and simultaneously contribute to impaired well-being [5].

The few existing studies on the relation between climate change knowledge and anxiety have produced inconsistent findings, reporting either positive or negative associations (e.g., [16,17]). A recent meta-analysis comprising nine primary studies found no significant overall association between climate change knowledge and anxiety, but indicated that the underlying effects are quite heterogeneous [5]. These mixed findings may reflect important limitations in prior research. Several studies relied on self-reported rather than objectively assessed knowledge (e.g., [18]), which is problematic because perceived and objective knowledge have been shown to relate differently to climate change perceptions [14]. For example, a study showed that only objectively assessed, but not perceived, climate change knowledge was positively related to climate change risk perceptions, which is closely linked to climate change anxiety [14,19].

Additionally, previous studies have not systematically differentiated between climate change anxiety [20] and climate change worry [21]. Climate change anxiety reflects a more clinically significant anxious response [20] and is accompanied by cognitive (e.g., difficulties concentrating due to climate change) and functional (e.g., difficulties completing work tasks due to climate change) impairments. It is commonly assessed with the validated and widely used Climate Change Anxiety Scale (CCAS [20]). Observed mean levels on this scale are typically relatively low, suggesting that more severe forms of climate change anxiety may be less common [5].

In contrast, climate change worry is generally conceptualized as a more common and less impairing response than climate change anxiety that may motivate climate action [5]. Although worrying about climate change can be considered a cognitive component of climate change anxiety, the two related constructs are conceptually and empirically distinct and differ in their prevalence and predictors. For example, in an online survey of the UK public, individual differences such as environmental values and nature relatedness were differently related to climate change anxiety, as measured with the CCAS, and climate change worry assessed with a single item (i.e., How worried are you about climate change [11]). Distinguishing between these outcomes is therefore essential for determining whether climate change knowledge primarily elicits adaptive worry or may also contribute to more clinical forms of climate change anxiety that impair functioning.

Furthermore, previous research on climate change knowledge and anxiety has largely focused on between-person relations, using cross-sectional data. Such designs reveal between-person associations (e.g., individuals who generally know more about climate change than others report generally higher levels of climate change anxiety). However, cross-sectional data cannot reveal within-person associations (e.g., when individuals can retrieve more knowledge about climate change than usual at a certain point in time, they are likely to experience more climate change anxiety). This is unfortunate, because emotional responses to climate change have been shown to vary substantially within individuals over time (e.g., [22]). Climate change knowledge may fluctuate as well as individuals continuously acquire new information while also forgetting previously learned content [23]. Therefore, the goal of our study was to advance previous cross-sectional research by exploring associations between climate change knowledge and climate change anxiety and worry, respectively, at both the between-person and within-person levels. To adequately disentangle associations at these levels, we used a study design with repeated measures. Importantly, our focus was on concurrent within-person associations (i.e., at the same time point) rather than dynamic relations from one time point to the next.

## 2 The present study

To address the aforementioned gaps, we collected data on climate change knowledge, assessed with two different, validated knowledge tests, and climate change anxiety and worry across four measurement waves with 3-month time lags, comprising 1,152 adults in Germany. We analyzed the data using mixed effects models which allowed us to estimate relations at both the between- and within-person levels.

Although there are theoretical arguments that greater climate change knowledge may be related to lower feelings of uncertainty and higher perceived control, thereby mitigating climate change anxiety [17,24], we expect that climate change knowledge is positively related to climate change anxiety and worry. Theorizing and empirical research on risk perceptions suggest that greater knowledge about climate change is associated with higher perceived risks [14], which, in turn, may be related to feelings of anxiety. Specifically, greater knowledge may lead individuals to appraise climate change as a severe threat, as they better understand both the complexity of coping with it and the potentially detrimental consequences of inaction [25]. Consistent with this reasoning, prior studies have found positive associations between climate change knowledge and risk judgements [26], climate change anxiety as measured with the CCAS [16], and climate change worry [27]. To explore differences between more clinical climate change anxiety and more adaptive climate change worry, we propose separate hypotheses for the two constructs across levels of analysis. Based on prior cross-sectional findings, and given that no theorizing exists that suggests otherwise, we expected similar associations at both levels of analysis.

*Hypothesis 1*: Climate change knowledge is positively related to climate change worry at both the between-person and within-person levels of analysis.

*Hypothesis 2*: Climate change knowledge is positively related to climate change anxiety at both the between-person and within-person levels of analysis.

## 3 Materials and method

### 3.1 Transparency, ethical considerations, and open science

To enable the estimation of between-person and concurrent within-person effects, we collected data using a repeated-measures design. Data for the present study were collected as part of a larger data collection effort spanning ten measurement waves between August 2022 and June 2024. For practical reasons (e.g., survey length), variables relevant for the present study (i.e., climate change knowledge, climate change worry, and climate change anxiety) were only included from Time (T) 7 to T10. Several other studies based on this data collection have been published but used non-overlapping variables [28], [29], [30], [31], [32], [33], [34], [35], [36], [37], [38], [39], [40]. A data transparency table is available in the online supplemental materials at the Open Science Framework (https://osf.io/zdauc). The study was approved by the ethical advisory board of Leipzig University (No. 2023.08.01_eb_vv_7, Study Title: Environmental Sustainability at Work) and informed consent was obtained from all participants. We provide statistical code and data to reproduce our analyses in the online Supplemental Materials.

### 3.2 Data collection procedure

We commissioned the ISO 20252:2019 certified online panel company Norstat to collect the data. The measurement waves relevant for the present study (i.e., T7-T10) were separated by 3-month time lags and were collected between September 2023 and June 2024. For the sake of simplicity, these measurement waves used for the current study are referred to as T1–T4 hereafter. At T1, 1,688 participants provided complete data on our substantial variables. We excluded 536 participants who provided incorrect answers to two forced response items (e.g., “Please select the answer option ‘very often’ for this item”), which were included to identify careless responders [41]. The final sample consisted of 1,152 participants, with the number of participants providing complete data varying between measurement waves (i.e., *n* = 890 at T2, *n* = 762 at T3, *n* = 663 at T4). To explore the possibility of systematic attrition, we compared descriptive statistics of demographic characteristics and substantive variables at T1 between participants with complete data across waves (n = 663) and participants with partial data across waves (n = 489). Table 1 presents the results of these difference tests and shows that complete responders were slightly older than incomplete responders. In addition, we estimated a binary logistic regression model that included demographic and substantive variables as predictors of response completeness. The model explained only a very small share of variance in completeness (Cox-Snell Pseudo *R*^2^ = 0.0212). This suggests that systematic dropout was not a major concern in our study (see [42]). Table 1 further presents descriptive statistics of demographic characteristics and T1 values of substantive variables for the overall sample (i.e., all participants that were included in the main analysis, including both complete and incomplete responders).

**Table 1:** Descriptive statistics of demographic characteristics and substantive variables at T1 and comparisons of complete and incomplete responders*.*

	T1-4 Incomplete *n* = 489	T1-4 Complete *n* = 663	*p*-value	Overall Sample *n* = 1,152
**Age (Years)**				
Mean (*SD*)	47.3 (12.3)	50.0 (11.0)	<0.001	48.9 (11.6)
Median [Min, Max]	48.0 [18.0, 72.0]	52.0 [19.0, 85.0]		50.0 [18.0, 85.0]
**Gender**				
Male	216 (44.2%)	321 (48.4%)	0.174	537 (46.6%)
Female	272 (55.6%)	341 (51.4%)		613 (53.2%)
Missing	1 (0.2%)	1 (0.2%)		2 (0.2%)
**Education**				
No qualification	1 (0.2%)	0 (0%)	0.620	1 (0.1%)
Lower Secondary School	24 (4.9%)	39 (5.9%)		63 (5.5%)
Intermediate Secondary School	159 (32.5%)	202 (30.5%)		361 (31.3%)
Upper Secondary School	133 (27.2%)	176 (26.5%)		309 (26.8%)
College/University	165 (33.7%)	238 (35.9%)		403 (35.0%)
Missing	7 (1.4%)	8 (1.2%)		15 (1.3%)
**T1 Climate Change Worry**				
Mean (*SD*)	3.32 (1.13)	3.35 (1.09)	0.616	3.34 (1.10)
Median [Min, Max]	3.25 [1.00, 5.00]	3.25 [1.00, 5.00]		3.25 [1.00, 5.00]
**T1 Climate Change Anxiety**				
Mean (*SD*)	1.75 (0.976)	1.71 (0.936)	0.441	1.72 (0.953)
Median [Min, Max]	1.38 [1.00, 6.00]	1.31 [1.00, 5.62]		1.31 [1.00, 6.00]
**T1 Climate Change Knowledge (Geiger et al., 2019)**				
Mean (*SD*)	4.13 (0.990)	4.24 (0.926)	0.056	4.20 (0.955)
Median [Min, Max]	4.00 [0, 5.00]	4.00 [0, 5.00]		4.00 [0, 5.00]
**T1 Climate Change Knowledge (Libarkin et al., 2018)**				
Mean (*SD*)	9.57 (3.11)	9.51 (3.10)	0.724	9.53 (3.10)
Median [Min, Max]	9.00 [2.00, 19.0]	10.0 [2.00, 18.0]		9.00 [2.00, 19.0]

*Note.* T1–T4 Incomplete Responders = All participants who provided at least partial data on the substantive variables across T1–T4. T1–T4 Complete Responders = All participants who provided complete data on the substantive variables across T1–T4. Overall Sample = All participants that were included in the main analysis, including both complete and incomplete responders. The *p*-values refer to comparisons between complete and incomplete responders using *t*-tests and Chi-square difference tests.

### 3.3 Measures

#### 3.3.1 Climate change knowledge

To check the robustness of our findings, we used two different, validated tests to assess climate change knowledge. First, we used the climate change subtest of the environmental knowledge test developed by Geiger et al. [13]. The climate change subtest consists of five single choice questions with four answer options each and focuses on various aspects of climate change (e.g., “Which of the following phenomena has been the main cause of global warming over the last 20 years?”). An overall knowledge score was built by summarizing the number of correct answers (ranging from 0 to 5). Second, we used the climate change understanding test developed by Libarkin et al. [12]. This test consists of 17 single choice questions with three to four answer options each and four multiple choice questions with four answer options each, of which two to four were correct. An overall knowledge score was built by summarizing the number of correct answers (ranging from 0 to 21).

#### 3.3.2 Climate change worry

Building on frequently used single item measures of climate change worry [11,43], we developed four items to assess climate change worry. The items are “How worried are you personally about climate change?” “How great is your personal worry regarding the impacts of climate change?” “To what extent does climate change worry you?” and “How worried are you in view of the possible consequences of climate change?” Items were rated on a five-point scale ranging from 1 (*not at all worried*) to 5 (*extremely worried*). The reliability of this scale was good (α_mean_ = .982, α_range_ = .980–.983, ω_mean_ = .982, ω_range_ = .980–.984).

#### 3.3.3 Climate change anxiety

We used the validated German translation of the climate change anxiety scale [20,44]. The scale comprises 13 items with a response scale ranging from 1 (*strongly disagree*) to 7 (*strongly agree*). The scale has two dimensions, namely cognitive-emotional impairment (e.g., “Thinking about climate change makes it difficult for me to concentrate”) and functional impairment (e.g., “My concerns about climate change interfere with my ability to get work or school assignments done”). This two-dimensional structure was not replicated in the German validation study of the scale [44]. However, in the present study, a confirmatory factors analysis (CFA) indicated that a three-factor model including climate change worry and the two dimensions of climate change anxiety fit the data well (χ^2^(116) = 855.144, *p* < .001; CFI = 0.914; RMSEA = 0.074; SRMR = 0.053) and better than a two-factor model where climate change anxiety was introduced as one factor along with climate change worry (χ^2^(118) = 1178.166, *p* < .001; CFI = 0.877; RMSEA = 0.088; SRMR = 0.057). Therefore, we report results for each climate change anxiety dimension separately in addition to the combined climate change anxiety score. We further specified a one-factor model where all items representing climate change anxiety and worry loaded on a single factor. The model indicated poor fit, suggesting that climate change anxiety and worry are distinct constructs (χ^2^(119) = 4227.108, *p* < .001; CFI = 0.522; RMSEA = 0.173; SRMR = 0.157). Reliability for climate change anxiety was good (overall climate change anxiety: α_mean_ = .952, α_range_ = .948–.956, ω_mean_ = .951, ω_range_ = .947–.956; cognitive-emotional impairment: α_mean_ = .917, α_range_ = .911–.924, ω_mean_ = .918, ω_range_ = .911–.925; functional impairment: α_mean_ = .936, α_range_ = .931–.940, ω_mean_ = .934, ω_range_ = .928–.939).

### 3.4 Analytical strategy

To test our hypotheses, we specified linear mixed effects models with the lme4 package [45] in R [46] version 4.5.0. Such models allow for the estimation of both between- and within-person associations across all measurement points. We fitted four models where climate change worry, overall climate change anxiety, and the two subdimensions of climate change anxiety were each predicted by climate change knowledge at the between- and within-person levels. This was done for both knowledge tests separately. Additionally, the models included a random intercept for each respondent. Between-person components were operationalized as each person's mean score across all measurement occasions, and within-person components as the deviation of each observation from the respective person mean [47]. We used a restricted maximum likelihood estimator [45]. Given the notable correlation between climate change worry and climate change anxiety (i.e., *r* = .11 at the within-person level and *r* = .44 at the between-person level), we also tested models where we controlled for the effects of climate change anxiety on climate change worry, and vice versa.

## 4 Results

**Fig. 1:**
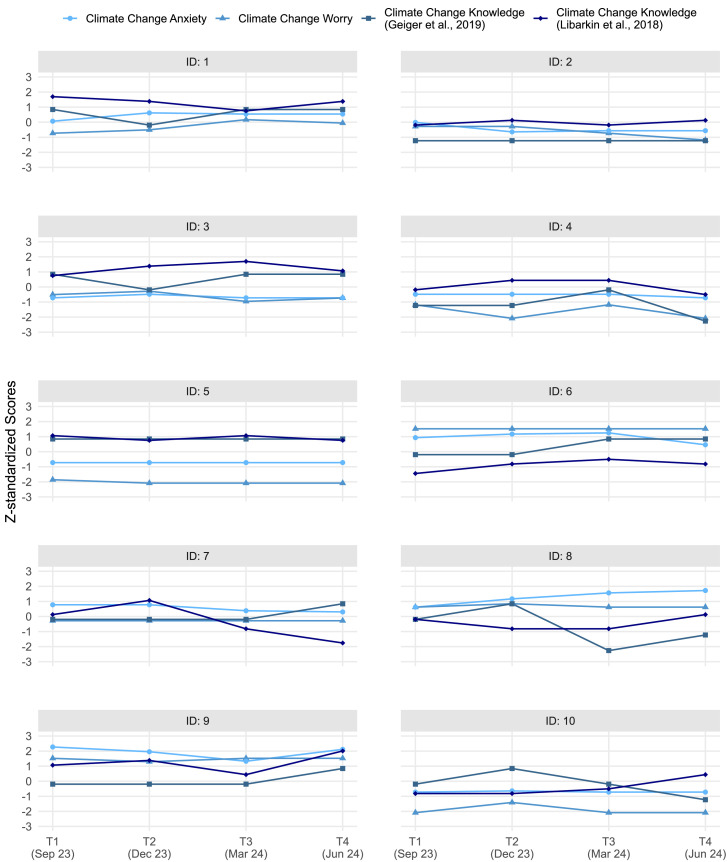
*Z-standardized scores of substantive variables across measurement waves for n = 10 randomly selected complete responders in the sample*.

**Fig. 2:**
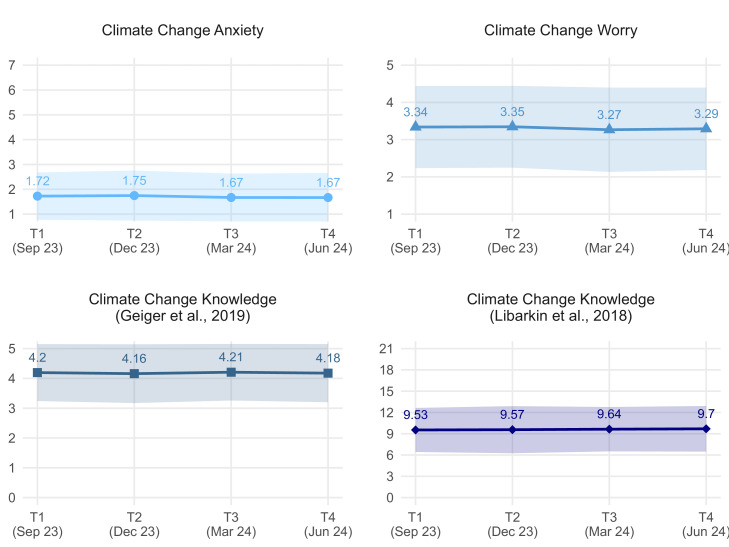
*Mean Levels of Substantive Variables Across Measurement Waves.* Climate change anxiety was measured on a 1–7 scale; climate change worry was measured on a 1–5 scale; climate change knowledge reflects the number of correct items (Geiger et al., 2019: range 0–5; Libarkin et al., 2018: range 0–21). Shaded areas represent ± 1 standard deviation. Repeated measures ANOVAs revealed no statistically significant change across measurement waves for climate change anxiety (*F*(3, 2019) = 2.48, *p* = .060); climate change knowledge as measured by Geiger et al., 2019 (*F*(3, 1998) = 1.37, *p* = .252); or climate change knowledge as measured by Libarkin et al., 2018 (*F*(3, 1986) = 1.54, *p* = .201). Climate change worry varied significantly across waves (*F*(3, 2034) = 5.28, *p* = .001), but the effect size was very small (η^2^p = .008). Post-hoc pairwise comparisons (Bonferroni corrected) revealed that worry was significantly higher in September 2023 than in March 2024 (*p* = .007) and June 2024 (*p* = .008), while all other wave comparisons were non-significant.

Descriptive statistics and correlations can be found in Table 2. A notable proportion of variance in our substantive variables resided at the within-person level (i.e., 13% for climate change worry, 23% for climate change anxiety, 47% for climate change knowledge as measured with the test by Geiger et al. [14], and 31% for climate change knowledge as measured with the test by Libarkin et al. [13]). To illustrate this variation within individuals, Fig. 1 displays the values of substantive variables across measurement waves for n = 10 randomly selected complete responders. To illustrate changes at the between-person level, Fig. 2 displays mean scores on the substantive variables across the four measurement waves. Overall, mean levels remained stable across waves, with no clear increasing or decreasing trend. Mean levels of climate change anxiety were low on average, whereas climate change worry was at a moderate level. For the climate change knowledge tests, participants answered a high proportion of items correctly on the test by Geiger et al. [13], whereas the number of correct answers was around the midpoint for the test by Libarkin et al. [12].

**Table 2:** Descriptive statistics and correlations*.*

	Variable	ICC1	*M*	*SD*	1.	2.	3.	4.	5.	6.	7.	8.	9.
1.	Age	**-**			-	-.06	-.19^⁎⁎^	-.10*	-.07*	-.12*	.05	-.03	.00
2.	Gender	**-**				-	-.13	.06	.09	.02	.15	-.20	-.30
3.	Educational level	**-**					-	.07	.06	.07	.02	.26	.31
4.	Climate change anxiety	.763	1.70	0.98				-	.99^⁎⁎^	.97^⁎⁎^	.44^⁎⁎^	-.10*	-.17^⁎⁎^
5.	Climate change anxiety: Cognitive impairment	.747	1.78	1.02				.94^⁎⁎^	-	.92^⁎⁎^	.45^⁎⁎^	-.12*	-.18^⁎⁎^
6.	Climate change anxiety: Functional impairment	.707	1.59	1.01				.86^⁎⁎^	.62^⁎⁎^	-	.40^⁎⁎^	-.08	-.14^⁎⁎^
7.	Climate change worry	.870	3.31	1.11				.11*	.12^⁎⁎^	.08*	-	.21^⁎⁎^	.09*
8.	Climate change knowledge Geiger et al.	.530	4.19	0.97				-.01	-.01	-.02	.04*	-	.65^⁎⁎^
9.	Climate change knowledge Libarkin et al.	.685	9.60	3.19				-.01	-.03	.01	.00	.03	-

*Note.* ICC1 = Intraclass Correlation Coefficient 1. Between-person correlations represent correlations between individuals’ average levels across the four measurement waves and are shown above the diagonal. Within-person correlations represent correlations between variables as they co-vary within individuals and are shown below the diagonal*. n* (between) = 1,135; *n* (within) *=* 3,645. The total number of between-person subjects is slightly lower than in the focal analyses because correlations were calculated using listwise deletion to resolve convergence issues. *p < .05, **p < .001.

Table 3 summarizes the results of the focal mixed-effects models. In support of Hypothesis 1, climate change knowledge was positively related to climate change worry at the between-person level. This relation emerged for both knowledge tests (test by Geiger: *B* = 0.22, *SE* = 0.04, *p* < .001, *B*_std_ = 0.16; test by Libarkin: *B* = 0.03, *SE* = 0.01, *p* = .004, *B*_std_ = 0.08). According to current correlational effect size benchmarks [48], these effects can be considered as small.

**Table 3:** Summary of mixed-effects regression models.

(a) Knowledge test by Geiger et al. [14]	CC Worry			CC Anxiety			CC Anxiety Cognitive Impairment			CC Anxiety Functional Impairment		
Predictors	*B*	*SE*	*p*	*B*	*SE*	*p*	*B*	*SE*	*p*	*B*	*SE*	*p*
Intercept	2.37	0.16	**<.001**	2.16	0.14	**<.001**	2.29	0.14	**<.001**	1.93	0.14	**<.001**
Knowledge - Between	0.22	0.04	**<.001**	-0.11	0.03	**.001**	-0.12	0.03	**<.001**	-0.08	0.03	**.014**
Knowledge - Within	0.03	0.01	**.011**	-0.01	0.01	.454	-0.01	0.02	.523	-0.01	0.02	.462

Marginal *R*^2^	0.026			0.007			0.009			0.004		


(b) Knowledge test by Libarkin et al. [13]	CC Worry			CC Anxiety			CC Anxiety Cognitive Impairment			CC Anxiety Functional Impairment		
Predictors	*B*	*SE*	*p*	*B*	*SE*	*p*	*B*	*SE*	*p*	*B*	*SE*	*p*
Intercept	3.01	0.11	**<.001**	2.17	0.09	**<.001**	2.29	0.10	**<.001**	1.98	0.09	**<.001**
Knowledge - Between	0.03	0.01	**.004**	-0.05	0.01	**<.001**	-0.05	0.01	**<.001**	-0.04	0.01	**<.001**
Knowledge - Within	-0.00	0.00	.949	-0.00	0.01	.495	-0.01	0.01	.191	0.00	0.01	.679

Marginal *R*^2^	0.006			0.019			0.021			0.012		


*Note.* CC = climate change*. n* (between) = 1,152.; *n* (within) = 3,696. Nakagawa et al.’s [49] pseudo marginal *R^2^* estimates are reported.

At the within-person level, however, only climate change knowledge as measured with the test by Geiger was positively related to climate change worry (*B* = 0.03, *SE* = 0.01, *p* = .011, *B*_std_ = 0.02), not climate change knowledge as measured with the test by Libarkin (*B* < 0.001, *SE* = 0.00, *p* = .949, *B*_std_ < 0.001). Within-person effects are typically smaller in magnitude than between-person effects in multilevel models (e.g., [50]). Results remained virtually unchanged when controlling for the effects of climate change anxiety on climate change worry (see Table 4).

**Table 4:** Summary of mixed-effects regression models with control for worry/anxiety.

**(a) Knowledge test by Geiger et al.** [14]	**CC Worry**			**CC Anxiety**			**CC Anxiety** **Cognitive Impairment**			**CC Anxiety** **Functional Impairment**		
Predictors	*B*	*SE*	*p*	*B*	*SE*	*p*	*B*	*SE*	*p*	*B*	*SE*	*p*
Intercept	1.26	0.16	**<.001**	1.26	0.13	**<.001**	1.34	0.14	**<.001**	1.14	0.14	**<.001**
Knowledge - Between	0.28	0.03	**<.001**	-0.19	0.03	**<.001**	-0.21	0.03	**<.001**	-0.16	0.03	**<.001**
Knowledge - Within	0.03	0.01	**.008**	-0.01	0.01	.295	-0.01	0.02	.339	-0.02	0.02	.350
CC anxiety - Between	0.51	0.03	**<.001**									
CC anxiety - Within	0.10	0.02	**<.001**									
CC worry - Between				0.38	0.02	**<.001**	0.40	0.02	**<.001**	0.34	0.02	**<.001**
CC worry - Within				0.14	0.02	**<.001**	0.16	0.03	**<.001**	0.11	0.03	**<.001**

Marginal *R*^2^	0.198			0.170			0.179			0.124		


(b) Knowledge test by Libarkin et al. [13]	CC Worry			CC Anxiety			CC Anxiety Cognitive Impairment			CC Anxiety Functional Impairment		
Predictors	*B*	*SE*	*p*	*B*	*SE*	*p*	*B*	*SE*	*p*	*B*	*SE*	*p*
Intercept	1.89	0.12	**<.001**	1.07	0.11	**<.001**	1.11	0.11	**<.001**	1.00	0.11	**<.001**
Knowledge - Between	0.06	0.01	**<.001**	-0.06	0.01	**<.001**	-0.06	0.01	**<.001**	-0.05	0.01	**<.001**
Knowledge - Within	0.00	0.00	.989	-0.00	0.01	.497	-0.01	0.01	.191	0.00	0.01	.675
CC anxiety - Between	0.51	0.03	**<.001**									
CC anxiety - Within	0.10	0.02	**<.001**									
CC worry - Between				0.37	0.02	**<.001**	0.39	0.02	**<.001**	0.33	0.02	**<.001**
CC worry - Within				0.14	0.02	**<.001**	0.16	0.03	**<.001**	0.10	0.03	**<.001**

Marginal *R*^2^	0.176			0.176			0.185			0.129		

*Note.* CC = climate change. *n* (between) = 1,152.; *n* (within) = 3,696. Nakagawa et al.’s [49] pseudo marginal *R^2^* estimates are reported.

Contrary to predictions of Hypothesis 2, climate change knowledge was negatively related to climate change anxiety at the between-person level for both knowledge tests (test by Geiger: *B* = -0.11, *SE* = 0.03, *p* = .001, *B*_std_ = -0.09; test by Libarkin: *B* = -0.05, *SE* = 0.01, *p* < .001, *B*_std_ = -0.14). This pattern also emerged for the two subdimensions of climate change anxiety (cognitive impairment: test by Geiger: *B* = -0.12, *SE* = 0.03, *p* < .001, *B*_std_ = -0,06; test by Libarkin: *B* = -0.05, *SE* = 0.01, *p* < .001, *B*_std_ = -0.14; functional impairment: test by Geiger: *B* = -0.08, *SE* = 0.03, *p* = .014, *B*_std_ = -0.06; test by Libarkin: *B* = -0.04, *SE* = 0.01, *p* < .001, *B*_std_ = -0.11). These represent small effects [48].

In contrast, at the within-person level, climate change knowledge was not significantly related to climate change anxiety (test by Geiger: *B* = -0.01, *SE* = 0.01, *p* = .454, *B*_std_ < 0.001; test by Libarkin: *B* = -0.00, *SE* = 0.01, *p* = .495, *B*_std_ < 0.001) or its subdimensions (cognitive impairment: test by Geiger: *B* = -0.01, *SE* = 0.02, *p* = .523, *B*_std_ < 0.001; test by Libarkin: *B* = -0.01, *SE* = 0.01, *p* = .191, *B*_std_ = -0.01; functional impairment: test by Geiger: *B* = -0.01, *SE* = 0.02, *p* = .462, *B*_std_ < 0.001; test by Libarkin: *B* = 0.00, *SE* = 0.01, *p* = .679, *B*_std_ < 0.001). Results remained virtually unchanged when controlling for the effects of climate change worry on climate change anxiety (see Table 4).

### 4.1 Exploratory analysis

To examine whether age, gender, and educational level may confound the relations between climate change knowledge and climate change anxiety and worry, respectively, we conducted exploratory analyses including these variables as covariates in our mixed-effects models. The inclusion of the demographic variables did not meaningfully alter the pattern of our findings. Age was positively related to climate change worry but negatively related to climate change anxiety. Gender differences indicated that women reported higher levels of climate change worry, whereas no gender differences were observed for climate change anxiety. Educational level was unrelated to climate change worry, but individuals with a college education reported higher levels of climate change anxiety. These results were consistent across both knowledge tests. Full results of the exploratory analyses are available in the online Supplemental Materials.

## 5 Discussion

Analyzing data across four measurement waves from 1,152 adults in Germany using mixed-effects models, we found that climate change knowledge was positively associated with climate change worry but negatively associated with climate change anxiety at the between-person level. These effects were robust across two different tests of climate change knowledge, held for both subdimensions of climate change anxiety, and remained unchanged when mutually controlling for worry and anxiety, respectively, and demographic variables. This indicates that individuals with greater knowledge about climate change tend to report higher levels of climate change worry, while being less likely to experience clinically relevant and functionally impairing levels of climate change anxiety. According to current correlational effect size benchmarks [48], these effects can be considered as small.

### 5.1 Theoretical and practical implications

These findings support, and partially reconcile, previously contrasting theoretical perspectives and empirical findings suggesting that, on the one hand, higher knowledge is associated with lower uncertainty and anxiety [17,24], and, on the other hand, higher risk and threat perceptions [14,16], thereby increasing anxiety. Our results imply that earlier mixed findings may partly stem from a lack of conceptual distinction between maladaptive, clinically relevant anxiety and more adaptive forms of worry about climate change. When these related constructs are disentangled, greater climate change knowledge appears to be associated with higher climate change worry but lower anxiety. Considered through the lens of stress and coping theory [25], climate change knowledge may amplify threat appraisal, leading individuals to worry about climate change, while simultaneously enhancing coping appraisal by providing clearer information about climate change mitigation and adaptation.

With the exception of a positive within-person association between climate change knowledge measured with the test by Geiger et al. [13] and climate change worry, we did not observe significant within-person relations. One possible explanation is that climate change knowledge shapes relatively stable cognitive appraisals of climate change, whereas short-term fluctuations in climate change worry and anxiety might depend on contextual and other personal factors. These may include salient climate-related events such as episodes of extreme weather [52] as well as momentary stress levels [53].

From a practical perspective, our results suggest that disseminating climate change knowledge carries no downside. On the contrary, greater knowledge may both be related to more adaptive worry, which can motivate pro-environmental behavior [21], and reduced clinically impairing levels of climate change anxiety [20]. This underscores the importance of systematically distributing climate change knowledge through schools, universities, mainstream media, and influential stakeholders, such as politicians and business leaders (for an overview of knowledge-based interventions, see [15]).

### 5.2 Limitations and future research

Our study has several limitations that future research should address. First, although we drew on theoretical arguments regarding the mechanisms linking climate change knowledge to climate change anxiety and worry (e.g., risk perceptions, perceived control), testing these mediators was beyond the scope of this study. In future studies, a dual-pathway model could integrate previously contradictory perspectives, suggesting that knowledge may increase risk perception, thereby fostering adaptive worry, while simultaneously reducing uncertainty and enhancing perceived control, thus lowering clinically relevant anxiety.

Second, although the two knowledge tests used in this study are validated and cover multiple climate-related domains, they primarily provide an overall knowledge score rather than distinct scores for different knowledge facets. However, prior research indicates that different knowledge contents may relate differently to climate change perceptions [14]. For example, knowledge about the causes and consequences of climate change was positively related to climate change worry, whereas action-related knowledge showed no significant association [51]. Exploring these knowledge dimensions in more detail could yield nuanced insights into which types of knowledge are related to adaptive worry or clinical anxiety.

Third, the design of our study did not allow for ruling out potential seasonal effects. For example, increased salience of climate change during the summer months could potentially influence climate change knowledge as well as related anxiety and worry. Future research with longer observation periods and more measurement waves could further explore potential seasonal effects on climate change knowledge, anxiety, and worry.

## Declarations

• **Data Availability:** The online supplemental material, data, and analysis code are available at the Open Science Framework: https://osf.io/zdauc/
• **Ethics Approval Statement:** Data collection was approved by the ethics advisory board of Leipzig University (No. 2023.08.01_eb_vv_7, Study Title: Environmental Sustainability at Work).
• **Funding:** This research did not receive any specific grant from funding agencies in the public, commercial, or not-for-profit sectors.

## CRediT authorship contribution statement

**Clara Kühner:** Writing – original draft, Project administration, Formal analysis, Conceptualization. **Hannes Zacher:** Writing – review & editing, Supervision, Data curation, Conceptualization.

## Declaration of competing interest

The authors declare that they have no known competing financial interests or personal relationships that could have appeared to influence the work reported in this paper.
